# High-Dose Vitamin E Supplementation Can Alleviate the Negative Effect of Subacute Ruminal Acidosis in Dairy Cows

**DOI:** 10.3390/ani13030486

**Published:** 2023-01-31

**Authors:** Zibin Wu, Yongqing Guo, Jiahao Zhang, Ming Deng, Zhenyu Xian, Haoming Xiong, Dewu Liu, Baoli Sun

**Affiliations:** College of Animal Science, South China Agricultural University, Guangzhou 510642, China

**Keywords:** SARA, vitamin E, rumen microorganism, serum metabolome

## Abstract

**Simple Summary:**

This experiment was conducted to investigate the effects of a high-dose vitamin E (VE) supplementation on rumen microorganisms and the blood metabolites of subacute ruminal acidosis (SARA) induced by a high-grain diet in dairy cows, and to explore the mechanism of action through the combined analysis of the microbiome and metabolome. Our studies indicate that a high dose of VE can alleviate a series of adverse effects caused by SARA.

**Abstract:**

The aim of this trial was to assess whether the supplementation of vitamin E (VE) in high-concentrate diets could improve the fermentation and blood metabolism in the rumen of dairy cows, thereby modulating the degree of the subacute ruminal acidosis (SARA) response and improving the performance. Seven Holstein cows (four fitted with ruminal cannulas) were fed three diets (total mixed rations) during three successive periods (each lasted for 18 d): (1) the control diet (CON); (2) a high-grain (HG) diet, which was the control diet supplied with a 15% finely ground wheat diet (FGW); and (3) a high-VE diet (HGE), which was the control diet provided with a 15% FGW and 12,000 IU of VE/head per day. The results indicated that VE was able to alleviate the reduction in the dry matter intake (DMI) and milk fat yield in cows caused by HG diets. The supplementation of VE significantly reduced the levels of lipopolysaccharide (LPS), histamine (HIS), and the total volatile fatty acid (TVFA) in the rumen. The supplementation of VE observably increased the antioxidant capacity of the milk and plasma. In addition, VE markedly reduced the plasma levels of endotoxin, HIS, and pro-inflammatory factors. The supplementation of VE significantly enriched the differential metabolites of the purine metabolism, cysteine, methionine metabolism, and ABC transporter synthesis pathway in the serum. The supplementation of VE also significantly increased the relative abundance of *Succiniclasticum* and decreased the relative abundance of *Treponema,* thus reducing the production of TVFA in the rumen. In conclusion, considering that the cows in this trial had high ketone levels (BHBA > 2.3 mmol/L), we found that VE could improve the rumen fermentation and blood metabolism by modulating the relative abundance of rumen microorganisms, thereby mitigating a range of adverse effects caused by SARA.

## 1. Introduction

In recent years, with the increase in the demand for milk and its related products, the ruminant breeding industry has been greatly promoted. To improve the performance of dairy cows and gain more economic benefits, people have had to adopt the mode of high-concentrate feeding [1]. However, feeding a highly concentrated diet has increased the incidence of rumen acidosis, which has become an important factor restricting the improvement of the milk quality and the development of the dairy industry in China. Subacute rumen acidosis (SARA) is a common nutritional and metabolic condition in ruminants, which is caused by the excessive feeding of fermentable carbohydrates and insufficient dietary crude fiber [2]. Generally, SARA is considered to occur when the rumen pH is lower than 5.6 and the daily duration is more than 3 h [3]. SARA can remarkably reduce the rumen pH value, cause a large number of deaths and the disintegration of Gram-negative bacteria, and increase abnormal metabolites such as the organic acids, histamines, and endotoxins in the rumen. In addition, SARA can also cause a rumen microbial community disorder and damage the health of cows. In recent years, an increasing number of studies have confirmed that improving the antioxidant and immune capacity of the body is an important means of regulating the occurrence of SARA. For example, it is proposed that beet meal particles could relieve SARA by improving the antioxidant capacity of cows [4]. It found that sodium butyrate could alleviate SARA by reducing MDA and increasing the T-AOC levels in mammary tissue [5].

Vitamin E (VE), also known as tocopherol, is a fat-soluble vitamin that structurally belongs to the class of phenolic compounds in which the sixth hydroxyl group on the dihydropyran ring carries an active hydrogen atom that is the structural basis for the antioxidant capacity [6]. Studies have shown that dietary VE can scavenge free radicals, enhance antioxidant capacity and immunity, and improve the performance of cows. The study found that a VE supplementation in diets could reduce the MDA content in cells to a certain extent, improve the antioxidant enzyme activity, enhance the antioxidant capacity in the rumen and body of cows, and reduce the inflammatory response [7]. It found that VE could alleviate the rumen oxidation effect, improve the fermentation yield of cows, and change the availability of rumen energy, thus benefiting the growth of rumen microorganisms [8].

However, the regulation of SARA by VE has not been reported. Therefore, in the present study, we investigated whether VE has a positive effect on the rumen fermentation parameters, abnormal metabolites, microflora, and blood metabolism during SARA in cows. The results of this study may provide a theoretical basis and practical guidance for the pathogenesis of SARA and the rational utilization of diet.

## 2. Materials and Methods

### 2.1. Animals and Experimental Design

Seven multiparous Holstein dairy cows (63 ± 12 days in milk; 611 ± 53 kg BW), four of which were fitted with ruminal cannulas, were fed three diets (Table 1) during three successive periods (each period included the 14 days of the experiment period and the 4 days of the sampling period) in this experiment. In the first period, all cows were fed the control diets without wheat (CON); in the second period, the cows were fed a high-grain diet (HG), which is the control diet supplied with 15% fine ground wheat; and in the third period, the cows were fed a high-VE diet (HGE), which is the high-grain diet supplemented with 12,000 IU/head per day of VE [9]. Previous reports indicated that SARA was not instantly and easily reversible [10], making other experimental designs (such as a Latin square) problematic.

The experiment was conducted at Dinghu Wen’s Dairy Farm (Guangdong, China). The animals were housed individually in a separate enclosure (2.5 m × 2.5 m) with rubber mattresses throughout the study. The cows were fed with a total mixed ration (TMR) at 0700 and 1800 h, and drinking water was constantly available. The cows were milked at 0630 and 1730 h.

### 2.2. Feed Samples and Analysis

During d15–d17 of each period, the diets offered and refusals of individual cows were recorded daily to calculate the DMI. The samples taken from the daily diets and refusals were collected and stored at −20 °C for chemical composition analysis. All the samples were dried at 65 °C in a forced air oven (Model 2000; Experimental Mill, Beijing, China) for 48 h. The dried samples were ground to pass a 1 mm screen using a Wiley mill (standard model 4; Arthur H. Thomas Co., Philadelphia, PA, USA). The contents of dietary crude protein (CP) and ether extract (EE) were determined according to the method described by [11]; the neutral detergent fiber (NDF) and acid detergent fiber (ADF) content were determined using the ANKOM A-200i fiber analyzer according to the method of [12]; starch was hydrolyzed with anthracene by perchloric acid and determined by colorimetry on a Synergy H1 Microplate Reader.

### 2.3. Milk Collection and Analysis

The cows were milked twice daily at 0630 and 1730 h. The milk weights were recorded during the 3 d data collection period, and the milk samples were taken from days 15 to 16 of each experimental period. The milk samples were mixed in the morning and evening according to the milk yield and were stored in 2 tubes. One sample was treated with potassium dichromate and the LACTOSCAN LWA Milk Composition Analyzer (LACTOSCAN LWA, Guangzhou, China) was used to determine the milk fat percentage, milk protein percentage, lactose percentage, and total solids. The second tube was cryo-preserved at −20 °C and the milk urea nitrogen (MUN) concentrations were determined on a Synergy H1 enzyme-labeled analyzer using the coloration described by [13]. The total antioxidant capacity (T-AOC), superoxide dismutase (SOD), glutathione peroxidase (GSH-Px), and malondialdehyde (MDA) were determined using ELISA kits from the Beijing North Institute of Biotechnology and a Boten Synergy H1 ELISA analyzer.

### 2.4. Blood Samples and Analysis

The blood samples were collected via a tail venipuncture at 0 h, 3 h, and 6 h after the morning feeding on d17 of each period into vacutainers containing sodium heparin anticoagulant, non-pyrogenic sodium heparin tubes, and isolated gel coagulation tubes (Yuli Medical Instrument, Jiangsu China). The blood was centrifuged at 3500 r/min for 15 min to separate the plasma and serum and was divided into several aliquots, frozen at −20 °C for measurement. The plasma concentrations of glucose (GLU), triglycerides (TG), the total cholesterol (TC), non-esterified fatty acids (NEFA), β-hydroxybutyric acid (BHBA), and plasma urea nitrogen (PUN) were determined on a Beckman 5800 u automatic biochemistry instrument. The insulin and glucagon concentrations were analyzed on an XH6080 radioimmunoassay analyzer using a radioimmunoassay kit (Beijing North Institute of Biotechnology). The T-AOC, SOD, GSH-Px viability, and MDA content were determined using a kit (Beijing North Institute of Biotechnology) with a Burton Synergy H1 enzyme standard analyzer. The plasma concentrations of LPS, HIS, cytokine IL-1, IL-6, tumor necrosis factor (TNF-α), binding bead protein (Hp), serum amyloid A (SAA), C-reactive protein (CRP), and lipopolysaccharide-binding protein (LBP) were determined using a bovine specific ELISA kit (Northern Institute of Biotechnology, Beijing, China).

### 2.5. Rumen Fermentation Parameters

The rumen cranial, caudal, dorsal, caudal ventral, and caudal dorsal contents were collected from the rumen fistulas at 0 h before morning feeding and 3 h, 6 h, 9 h, and 12 h after morning feeding on d18 of each period, respectively, and were filtered by autoclaved four-layer mesh gauze with a size of 250 μm. The METTLER TOLEDO FE28-Standard pH meter was used to measure the pH value immediately. For the VFA analysis, a 20 mL filtered sample was put into a plastic bottle with 3 mL of 25% metaphosphoric acid and 3 mL of 0.6% 2-ethyl butyric acid (internal standard) and stored at −20 °C. The VFA concentration was determined as described by the Agilent 6890B gas chromatograph using gas chromatography [14]. The ammonia nitrogen (NH_3_-N) and lactic acid (LA) concentrations were measured using colorimetry on the Botten Synergy H1 enzyme conjugate analyzer. The LPS and HIS were determined by the ELISA kit (North Institute of Biotechnology, Beijing, China).

### 2.6. Metabolic Profile Analyses of Serum

The serum samples were analyzed using an LC-MS platform as described previously [15]. Subsequently, the samples were resuspended by vortexing with pre-chilled methanol and 0.1% formic acid, followed by incubation on ice for 5 min and centrifugation at 15,000× *g* for 20 min at 4 °C. After centrifugation, the supernatant was diluted to a final concentration of 53% methanol using LC-MS grade water. The sample was then transferred to an Eppendorf tube with a 0.22 μm filter membrane and centrifuged at 15,000× *g* for 20 min at 4 °C. Finally, the filtrate was injected into the LC-MS system for analysis. All LC-MS analyses were performed using a Vanquish UHPLC system (Thermo Fisher Scientific, Waltham, MA, USA) and an Orbitrap Q Exactive HF-X mass spectrometer (Thermo Fisher Scientific, Waltham, MA, USA). The method was as follows: the samples were injected onto a Hypersil Gold column (100 mm × 2.1 mm, 1.9 μm) using a linear gradient elution at a flow rate of 0.2 mL/min. The eluents for the positive polar mode were eluent A (0.1% formic acid) and eluent B (methanol), while the eluents for the negative polar mode were eluent A (5 mM ammonium acetate) and eluent B (methanol). The elution procedures were: 2% B for 15 min; 98% B for 12 min; 100% B for 14 min; 98% B for 14.1 min; and 2% B for 16 min. Finally, the Q Exactive HF-X mass spectrometer was operated in positive and negative polar modes with a spray voltage of 4 kV, a capillary temperature of 350 °C, a sheath gas flow rate of 35 arb, and an auxiliary gas flow rate of 10 arb. To perform the peak alignment, peak pickup, and quantification for each metabolite, we processed the metabolite data using Compound Discoverer 3.1 (CD 3.1, Thermo Fisher) software. Subsequently, multivariate statistical analysis was performed on the data using pareto scaling, and principal component analysis (PCA) was performed on the peaks extracted from the samples. The importance in projection (VIP) values of the PLS-DA variables were combined with the *p*-value from the t-tests to screen for significantly different metabolites. The screening criteria were as follows: VIP > 1.0, FC > 1.2 or FC < 0.833, and *p* < 0.05 [16]. In addition, the Kyoto Encyclopedia of Genes and Genomes (KEGG) database was used to analyze the functional characteristics and classification of the differential metabolites [17].

### 2.7. Microflora Analyses of Rumen

After the extraction of the rumen contents’ DNA by the CTAB method according to the manufacturer’s instructions, the concentration and quality of the DNA samples were determined by NanoDrop2000/2000C (Thermo, Waltham, MA USA). The 16SrRNA V4 region of the genomic DNA was amplified using Pyrobest DNA polymerase (Takara, DR500A) according to [18], with primer pairs designed as 515F (5′-GTGYCAGCMGCCGCGGTAA-3′) and 806R (5′-GGACTACNVGGGTWTCTAAT-3′). PCR amplification was performed using a Phusion^®^ High-Fidelity PCR Master Mix with a GC Buffer from New England Biolabs, and the PCR products were quality controlled and purified. The PCR products were sequenced by equimolar paired-end sequencing on the Illumina Novaseq 6000 platform (Personal Biotechnology Co., Ltd., Shanghai, China). The sequenced data were processed with QIIME (V 1.8.0) software, and valid sequences were aggregated into operational taxonomic units (OTUs) by UCLUST with a 97% similarity threshold [19]. The Basic Local Alignment Search Tool was used for a further taxonomic classification, and the OTUs were obtained [20]. The α- and β-diversity of the bacterial communities were calculated by the QIIME software and the vegan package in R software [21]. The function of the bacterial community was predicted through the PICRUST database [22]. Linear discriminant analysis (LDA) effect size (LEfSe) analysis was performed using an online tool, and LDA scores > 3 and *p* < 0.05 were selected as the thresholds.

### 2.8. Statistical Analysis

All data were analyzed using SAS (ver. 9.4, SAS Institute Inc., Cary, NC, USA). The normality of the data was tested using the UNIVARIATE procedure. The outliers were processed based on the absolute studentized residual values > 3. A statistical analysis of all data was performed using the GLM procedure. The model was used for data processing, including fixed effects for the forage treatment, fixed effects for the period, and random effects for one cow. Tukey adjustments were used for multiple comparisons. The experimental data were shown in the table by the means and standard error of the means (SEM). *p* < 0.05 meant a significant difference and *p* < 0.01 meant an extremely significant difference.

## 3. Results

### 3.1. Dry Matter Intake, Milk Yield, and Milk Composition

The DMI, milk yield, and milk composition during the HG diets are shown in Table 2. Compared to the CON, the cows fed the HG diet had a significantly lower DMI (21.42 vs. 19.40 kg/d; *p* < 0.01), 3.5 fat correct milk (3.5% FCM) (36.64 vs. 26.90 kg/d; *p* < 0.01), energy corrected milk (ECM) (36.04 vs. 27.96 kg/d; *p* = 0.001), feed efficiency (1.71 vs. 1.38; *p* = 0.003), milk fat yield (1.38 vs. 0.88 kg/d; *p* < 0.01), total solids yield in the milk (4.23 vs. 3.58 kg/d; *p* = 0.033), and MUN (13.68 vs. 11.30 mg/dL; *p* = 0.015). The HG diet also observably reduced the antioxidant indicators in the milk (T-AOC, SOD, CAT, and GSH-Px activity) and significantly increased the milk protein (3.12 vs. 3.31%, *p* = 0.007), lactose (4.53 vs. 4.78%; *p* = 0.012), and MDA content in the milk (5.01 vs. 7.98 nmol/mL; *p* < 0.01). Furthermore, the milk production was reduced by the HG diet, but the differences were not significant.

The effects of a VE supplementation on the DMI, milk yield, 3.5% FCM, ECM, and feed efficiency of cows are shown in Table 2. There was a trend towards a higher milk yield in cows in the HGE diet period compared to the HG diet period (29.38 vs. 32.85 kg/d; *p* = 0.238). However, the cows which received a supplementation had a significantly higher DMI (19.40 vs. 22.23 kg/d; *p* < 0.01), 3.5% FCM (26.90 vs. 32.94 kg/d; *p* = 0.009), ECM (27.96 vs. 33.29 kg/d; *p* = 0.018), and milk fat yield (0.88 vs. 1.16 kg/d; *p* < 0.01) compared to those which were fed the HG diet. VE also markedly increased the activity of T-AOC, CAT, SOD, and GSH-Px in the milk and decreased the content of MDA (Table 3).

### 3.2. Ruminal Fermentation Parameters

The ruminal fermentation parameters in the HG diet are illustrated in Table 4 and Table 5. The highest rumen pH (6.94 vs. 6.54; *p* = 0.009) and lowest rumen pH (5.99 vs. 5.52; *p* = 0.006) in the HG diet were lower than that in the CON diet. The rumen pH in the HG diet was significantly lower than the CON diet at 3 h and 6 h and was lower than 5.8 for 3 h, which indicated that the SARA model induced by increasing the FGW in the diet was successfully established in this study. Compared with the CON diet, the concentration of LPS and HIS was higher (*p* < 0.01), and the NH_3_-N concentration was significantly lower (*p* = 0.001) in the rumen of SARA cows induced by a high concentration. In addition, the total volatile fatty acid (TVFA), acetic acid, and butyric acid concentrations were markedly increased (*p* < 0.01; *p* < 0.01; *p* = 0.011, respectively), and the molar ratio of acetic acid was significantly reduced (*p* = 0.011) in the rumen of cows in the HG diet.

The effects of a VE supplementation on the fermentation parameters of the rumen of the cows are shown in Table 4 and Table 5. The highest pH, lowest pH, and mean pH of the rumen were higher after the supplementation of VE than in the HG diet, and the pH of the rumen increased at 3 h (5.70 vs. 5.83; *p* = 0.267) and 6 h (5.52 vs. 5.64; *p* = 0.387). The supplementation of VE also significantly reduced the concentration of LPS and HIS in the rumen (26.81 vs. 20.02 EU/mL; *p* < 0.01; 25.13 vs. 22.11 ng/mL; *p* < 0.01, respectively) and significantly increased the NH_3_-N concentration (8.36 vs. 11.67 mg/100 mL; *p* = 0.011). In addition, the TVFA (120.01 vs. 106.36 ng/uL; *p* = 0.006), acetic acid (66.60 vs. 60.50 ng/uL; *p* < 0.01), and butyric acid concentrations (19.89 vs. 14.44 ng/uL; *p* = 0.042) were significantly lower in the rumen of cows after a VE supplementation modulation compared to the HG diet.

### 3.3. Blood Indicators

Data on the effects of high-concentrate-induced SARA on the plasma biochemical, immunological, and antioxidant indices are given in Table 6, Table 7 and Table 8, respectively. The plasma GLU and UREA levels were markedly lower (*p* = 0.02; *p* < 0.01, respectively), however, the NEFA and BHBA concentrations were significantly higher (*p* = 0.011; *p* = 0.012, respectively) in the HG diet. The total cholesterol and insulin (INS) levels tended to decrease, but the difference was not significant (*p* > 0.05). The plasma levels of IL-1β, IL-6, TNF-α, Hp, CRP, SAA, LPS, and HIS were observably higher in the HG diet cows compared to the CON cows (*p* < 0.01). In addition, the plasma T-AOC, CAT, SOD, and GSH-Px viability were significantly lower (*p* < 0.01), while the MDA levels were significantly higher (*p* < 0.01) in HG diet cows.

Data on the effects of a VE supplementation on the plasma biochemical, immunological, and antioxidant parameters in cows are shown in Table 6, Table 7 and Table 8, respectively. The plasma GLU levels tended to increase in the HGE diet compared to the HG diet, but the difference was not significant (*p* = 0.673). The supplementation of VE significantly increased the plasma UREA levels (3.29 vs. 4.52 mmol/L; *p* < 0.01). VE also significantly increased the plasma SOD (269.00 vs. 304.59 U/mL; *p* = 0.001) and GSH-Px activity (868.88 vs. 1037.66 U/mL; *p* < 0.01). Compared with the HG diet, the supplementation of VE significantly reduced the levels of MDA (*p* = 0.007), IL-1β (*p* = 0.006), IL-6 (*p* < 0.01), TNF-α (*p* = 0.003), SAA (*p* = 0.013), LPS (*p* < 0.01), and HIS (*p* < 0.01).

### 3.4. LC/MS Analysis of Serum

**Figure 1:**
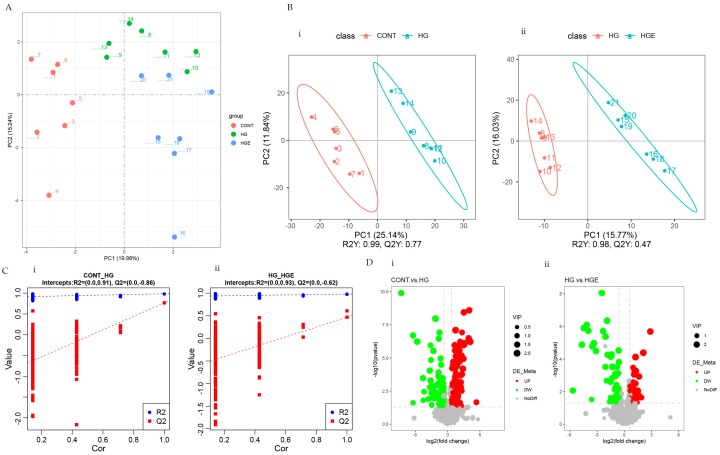
Effect of vitamin E on blood metabolism profile of subacute ruminal acidosis induced by high concentration. (**i**) CON-HG, (**ii**) HG-HGE. (**A**) Principal component analysis (PCA) scores plots, (**B**) projections to latent structures-discriminant analysis (PLS-DA) scores plots, (**C**) permutation test of the PLS-DA model, (**D**) volcano plots.

As shown in Figure 1A,B, there was marked clustering among the three phases based on the PCA and PLS-DA models. This result indicates that the distribution of the serum metabolites was significantly different among the CON, HG, and HGE diets. To avoid the transition fit of the OPLS-DA model, the alignment test was used for validation. The results indicated that the OPLS-DA model was stable, without overfitting, and could better describe the samples (Figure 1C).

OPLS-DA analysis was then performed to calculate the VIP values of each metabolite. The differential metabolites were screened by VIP > 1.0, FC > 1.2 or FC < 0.833, and *p* < 0.05. A total of 209 differential metabolites were screened in the CON-HG, of which 133 were significantly up-regulated and 76 were significantly down-regulated. The number up-regulated and down-regulated in the HG-HGE was 35 and 57, respectively (Figure 1D). The major differential metabolites can be seen in Table S1.

**Figure 2:**
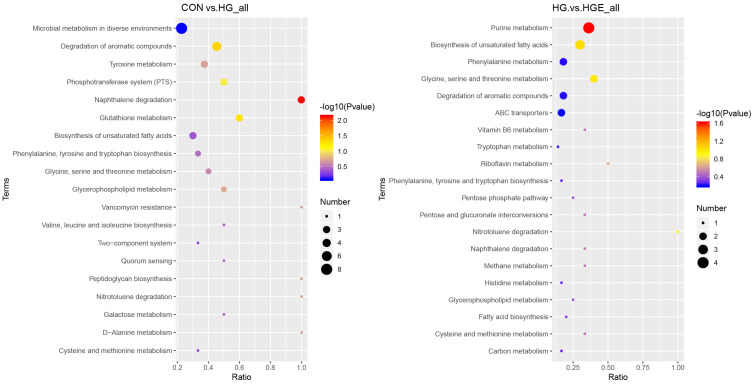
Functional annotation of differential metabolites. (**left**) CON-HG, (**right**) HG-HGE.

Based on the above results, it is necessary to further analyze these differential rumen metabolites. According to the pathway topology analysis, the metabolites of the differential metabolites in the CON-HG were significantly enriched in the naphthalene and aromatic compound degradation pathways, while the differential metabolites in the HG-HGE were significantly enriched in the purine metabolic pathway (Figure 2).

**Figure 3:**
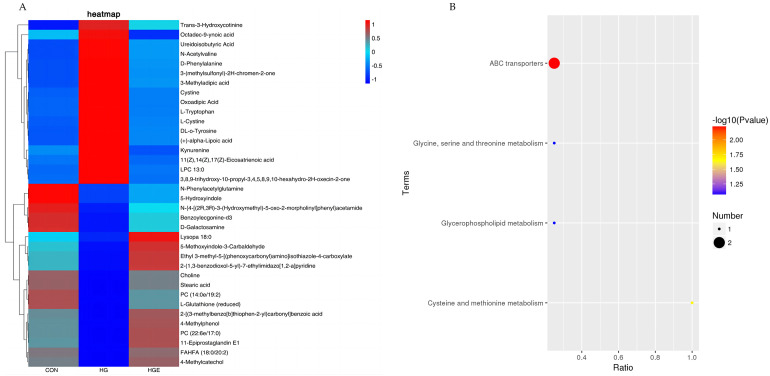
Analysis of common differential metabolite enrichment in CON-HG and HG-HGE. (**A**) Cluster heat map of common differential metabolites, (**B**) KEGG pathway enrichment map of common differential metabolites.

A total of 36 common differential metabolites were screened for the differential metabolites identified in the CON-HG and HG-HGE (Table S2), and the results of the cluster analysis are shown in Figure 3A. For these common differential metabolites, the KEGG enrichment analysis revealed, as is shown in Figure 3B, that Choline and L-Cystine were significantly enriched to the pathway ABC transporters, and L-cysteine was significantly enriched to the cysteine pathway and methionine metabolism (*p* < 0.05).

### 3.5. Richness, Diversity Estimates, and Rumen Bacteria Composition

**Figure 4:**
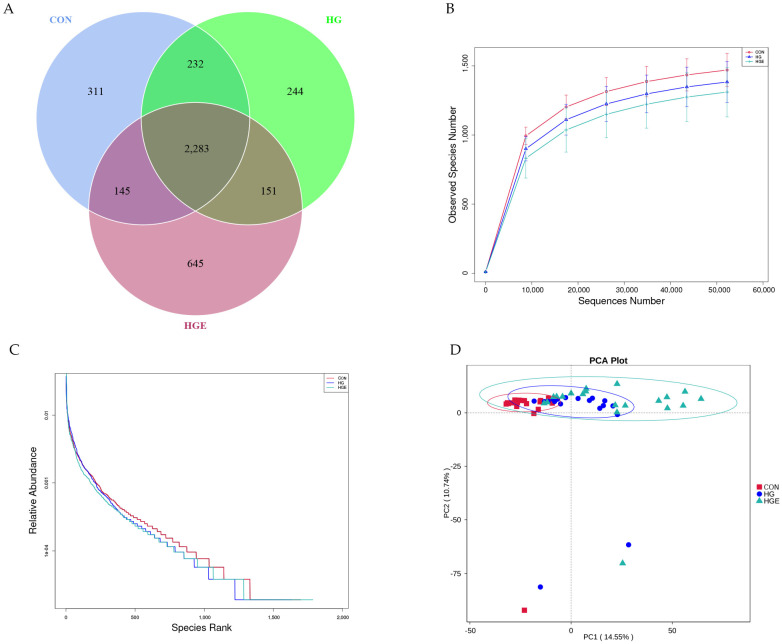
Ruminal microbial operational taxonomic. CON: basal diets without supplements; HG: high-grain diet; HGE: high-grain diet supplemented with 12,000 IU of vitamin E/head per day. (**A**) Venn diagram of ruminal bacterial OTUs. The number of unique OTUs was represented by the non-overlapped portion of the Venn diagram for each group. (**B**) Bacterial rarefaction curves based on OTUs were used to assess the depth of coverage for each sample. (**C**) Rank abundance curves based on bacterial OTUs were used to assess species richness and evenness in each treatment group. (**D**) Principal component analysis (PCA) of bacterial community structures of the ruminal microbiota in CON (red rectangle), HG (blue circles), and HGE (green triangle) diet groups.

The samples were sequenced by 16S rRNA to obtain 6,152,650 raw data, and a total of 3,824,044 valid data were obtained by quality control. The rarefaction curve was approximated to be smooth, indicating that the sequencing coverage was saturated (Figure 4B). As shown in Table 9, the Shannon index of the HG diet was significantly lower than that of the CON, and the Shannon and Simpson indices of the HGE diet were extremely significantly lower than that of the HG diet (*p* < 0.01). PCA analysis showed that the samples of the CON and HG diets were more concentrated, and the HGE diet could be separated (Figure 4D). Venn analysis showed (Figure 4A) that there were 311, 244, and 645 unique OTUs in the CON, HG, and HGE diets, respectively, for a total of 2283 OTUs. The MRPP analysis showed (Table 10) that the A values were all greater than 0, indicating that the differences during the three test periods were greater than the differences within the period and that there were significant differences in the rumen flora structure (*p* < 0.05).

**Figure 5:**
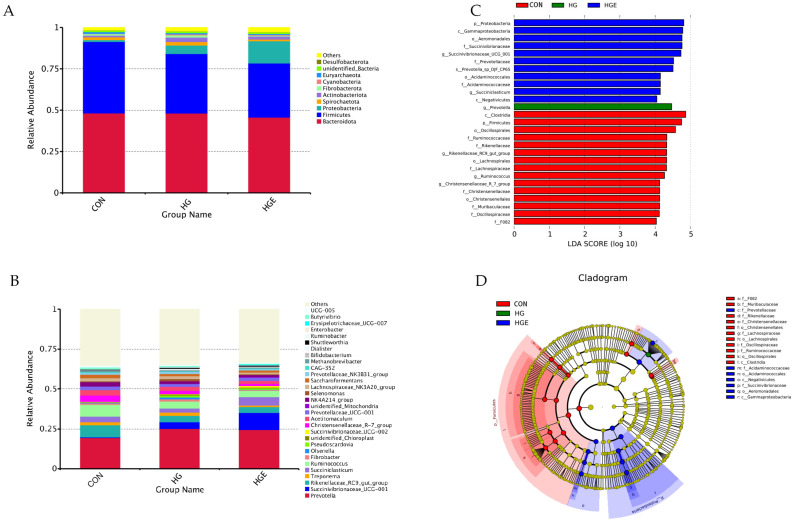
Effect of vitamin E on bacterial community relative abundance and lefse difference of subacute ruminal acidosis cows induced by high concentration at phylum and genus levels. The composition of rumen microflora at phylum (**A**) and genus (**B**) levels in cows. (**C**) Linear discriminant analysis (LDA) effect size (LEfSe) analyses of bacterial communities in cows based on the threshold of LDA score > 3. (**D**) Cladogram of the discovered biomarkers.

**Figure 6:**
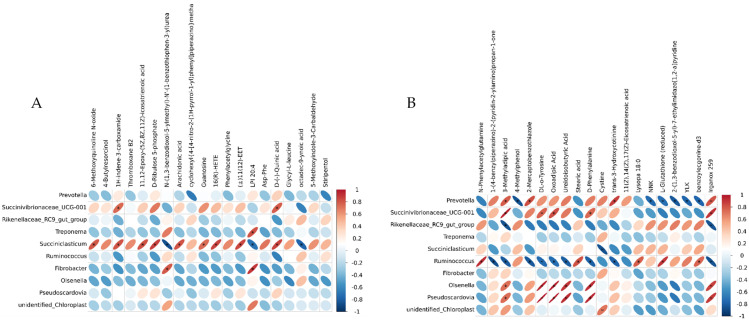
Correlation analysis between differential OTUs and metabolites. (**A**) CON-HG correlation analysis heat map. (**B**) HG-HGE correlation analysis heat map.

All the rumen bacterial reads were allocated to 41 phyla and 488 genera. *Bacteroidota*, *Firmicutes*, and *Proteobacteria* accounted for about 90% of the total flora. As shown in Figure 5 and Table S3, compared with the CON diet, the relative abundance of *Firmicutes* in the HG and HGE diets was significantly increased, and that of *Actinobacteriota* in the HG diet was significantly increased (*p* < 0.05). Compared with the HG diet, the relative abundance of *Proteobacteria* in the HGE diet was significantly up-regulated, while that of *Spirochaetota* was significantly down-regulated (*p* < 0.05). As shown in Figure 6 and Table S4, at the genus level, the top 10 genera with a high relative abundance are *Prevotella*, *Succinivibrionaceae_UCG-001*, *Rikenellaceae_RC9_gut_group*, *Treponema*, *Succiniclasticum*, *Ruminococcus*, *Fibrobacter*, *Olsenella*, *Pseudoscardovia*, and *unidentified_Chloroplast*. Compared with the CON diet, the relative abundance of *Succinivibrionaceae_UCG-001*, *Prevotella*, and *Pseudoscardovia* in the HG and HGE diets were significantly up-regulated, while the relative abundance of *Rikenellaceae_RC9_gut_group* and *Ruminococcus* were significantly down-regulated (*p* < 0.05). In addition, the relative abundance of *Treponema* and *Fibrobacter* in the HGE diet was significantly lower than that in the HG diet (*p* < 0.05), and the relative abundance of *Succiniclasticum* was significantly higher than that in the HG diet (*p* < 0.05).

### 3.6. Correlations between Blood Metabolome and Rumen Microbiome

The differential metabolites in the top 20 of the serum metabolomes (sorted by *p*-value from smallest to largest) and the differential genera in the top 10 of the rumen microbiomes (sorted by *p*-value from smallest to largest) were subjected to genus-level correlation analysis to achieve a more comprehensive understanding of the cow organism. Figure 6 shows the heat map based on the Pearson correlation coefficient for correlation analysis. Using |rho| ≥ 0.6 and *p* ≤ 0.05 as screening conditions, we could find that *Olsenella* and *Pseudoscardovia* were significantly and positively correlated with differential metabolites such as 3-methyladipic acid, DL-o-Tyrosine, oxoadipic acid, and D-Phenylalanine in the CON-HG, and the relative abundance of these genera was significantly higher in the high-concentrate feeding pattern. The relative abundance of *Ruminococcus* was significantly reduced in the high-concentrate feeding pattern, and it was significantly negatively correlated with differential metabolites, such as 3-methyladipic acid, DL-o-lipoic acid, oxoadipic Acid, and D-phenylalanine (Figure 6A). Arachidonic acid and D-ribulose 5-phosphate were significantly positively correlated with *Succiniclasticum* in the HG-HGE, while a high-dose VE modulation significantly increased the relative abundance of *Succiniclasticum* (Figure 6B).

## 4. Discussion

With the development of animal husbandry, many individuals are attempting to obtain better economic benefits by increasing the high concentration of grain in the ration of their cows’ food, but this may cause nutritional metabolic diseases such as rumen acidosis and ketosis. Since the blood BHBA concentrations of the cows in this trial were generally high, the following discussion is based on the evaluation of cows which were in a state of high ketogenic levels. In this study, we found that a VE supplementation in cows alleviated the reduction in the DMI and milk fat production in cows caused by high feeding levels, and that VE had an improved effect on increasing the antioxidant capacity in milk and blood, as well as reducing the rumen levels of LPS, HIS, and TVFA.

### 4.1. Dry Matter Intake, Milk Yield, and Milk Composition

The dry matter intake is an important factor affecting the performance of dairy cows. Malekkhahi et al. [23] found that the DMI of SARA cows induced by a high concentration was significantly reduced, which was similar to the results of this experiment. The decrease in the DMI of cows during SARA can be explained by the fact that in a low pH environment, the decreased absorption of VFA by the rumen epithelial cells leads to increased osmotic pressure in the rumen, a decreased rumen motility, and decreased fiber degradation, which ultimately affects the feed intake of cows [24]. A total of 3.5% FCM and ECM were positively correlated with the milk yield and milk fat yield. In this study, the 3.5% FCM, ECM, and feed utilization efficiency in the HG diet were significantly lower than those in the CON diet, indicating that the high-concentrate feeding mode changed the rumen fermentation mode due to its high proportion of rapidly degradable starch, thus reducing the energy utilization efficiency of the feed.

However, compared with an HG diet, a VE supplementation can significantly improve the DMI, 3.5% FCM, ECM, and feed utilization efficiency of cows, which may be related to the strong antioxidant capacity of VE, the improving the rumen microbial activity, promoting the synthesis and metabolism of other nutrients in vivo, and thus improving the feed nutrient utilization efficiency. In addition, there was an increasing trend in the milk yield in the HGE diet, but the difference was not significant, which might be related to the supplemental dose of VE.

Milk composition is an important index to measure the quality of dairy products. Aditya et al. [25] reported that SARA in cows leads to a reduced milk fat content and other researchers [26] have found that the milk protein and lactose contents were significantly increased in SARA dairy milk. In this trial, the milk fat production was significantly lower in the HG diet compared to the CON diet, which may be due to the cow’s energy intake not meeting the lactation requirements or to the reduced rumen fiber digestibility due to a lower DMI, which reduces the proportion of propionic acid–acetic acid in the rumen. A high concentrate induced a significant increase in the milk protein and lactose content and a significant decrease in the milk urea nitrogen content in the milk of SARA cows, probably due to the higher effective rumen carbohydrate content in a high concentrate induction, which facilitated the proliferation of rumen microorganisms and thus promoted the synthesis of rumen microbial proteins, resulting in a higher milk protein content and lower rumen NH_3_-N and blood UREA concentrations [27]. The propionic acid and lactic acid that are degraded by microorganisms in the rumen from high concentrations can be synthesized into glucose through the hepatic gluconeogenesis pathway, which eventually leads to elevated lactose. Oxidative stress is an important factor leading to immune dysfunction and an increased susceptibility to diseases. High concentrations of induced SARA reduce the antioxidant capacity in cow milk, which may be due to the low pH environment in the rumen leading to increased levels of LPS in vivo, altering the oxidative stress status in vivo [5]. Therefore, the SARA model is successfully constructed.

The supplementation of VE significantly increased the milk fat content and antioxidant capacity in milk compared with HG rations, probably due to the strong antioxidant properties of VE, which can improve the antioxidant capacity of the cow organism, reduce lipid peroxidation in the body, and reduce the damage to the organism and cells by SARA to some extent. The biohydrogenation of the rumen fat in cows is a complex process, and antioxidants help maintain the normal rumen hydrogenation pathway. Therefore, a VE supplementation can promote milk fat synthesis in cows.

### 4.2. Rumen Fermentation Parameters

The rumen fermentation parameters reflect the rule of rumen substance transformation and body health level. It is reported that the rumen pH was low, rumen TVFA, acetic acid, and butyric acid contents were significantly increased, and the acetic acid molar ratio was significantly decreased in SARA cows [28]. It was found that the contents of LPS and HIS in the rumen were significantly increased and NH_3_-N was significantly decreased when SARA occurred in cows [29]. In this experiment, compared with the CON diet, the rumen pH and the acetic acid molar ratio of SARA induced by a high concentration decreased, and the contents of TVFA, acetic acid, and butyric acid in SARA significantly increased. The ruminal pH showed the lowest pH at 6 h after feeding, which may be due to the rapid degradation of fermentable carbohydrates such as starch in the diet after feeding, producing a large number of volatile fatty acids and thus causing a decrease in the pH of the rumen fluid. However, as the content of fermentable carbohydrates decreases, the rate of volatile fatty acid production decreases, and together with the buffering effect of rumen epithelium and saliva, the pH of the rumen fluid gradually increases. In addition, SARA induced by high concentrate rations significantly increased the ruminal LPS and HIS and significantly decreased the ruminal NH_3_-N content, which may be due to the high starch content in high concentrate rations, which provides more energy to microorganisms, thus promoting the ruminal microbial proliferation, the utilization of ruminal NH_3_-N, and the decrease in the ruminal NH_3_-N concentration [30].

However, compared with a HG diet, a VE supplementation can alleviate the increase in the volatile fatty acid content in the rumen induced by high concentrate SARA, suggesting that the regulation of VE can improve the rumen fermentation pattern and reduce the production of volatile fatty acids in cows induced by high concentrate SARA, thus alleviating the adverse effects of SARA on the body. In addition, the supplementation of VE can alleviate LPS, and HIS production induced by a high concentrate, which may be due to the antioxidant property of VE, which can protect the bacterial plasma membrane in the rumen from a free radical attack, maintain bacterial integrity, and reduce the death of Gram-negative bacteria, thus reducing the production of LPS and HIS. VE also significantly increased the DMI of cows and increased the intake of protein and non-protein nitrogen in the feed, thus increasing the rumen NH_3_-N concentration.

### 4.3. Blood Indicators

The blood biochemical index is an important parameter to judge the physiological function of the body, which can reflect the metabolism and health status of the body. Xu et al. [31] showed that a high-concentration induction could reduce the concentrations of NEFA and BHBA in the blood of cows. Rodriguez et al. [32] reported that the UREA content in the plasma of cows was significantly decreased when SARA occurred. In this experiment, the concentration of BHBA in the blood of the control and treated cows was higher than 2.3 mmol/L, which may be due to the generally high proportion of concentrate in the diet during peak lactation and the cows being in a high milk production state, resulting in a negative energy balance for all cows in this experiment. In addition, the local climatic characteristics and differences in the feeding management on different dairy farms may also affect the concentration of BHBA in cow plasma. In general, the blood levels of beta-hydroxybutyric acid are considered to be the gold standard for diagnosing subclinical ketosis [33]. The current cutoff value of 1.2 mmol/L is utilized to differentiate between cows with and without subclinical ketosis [34]. Clinical ketosis generally involves much higher levels of BHB, about 3.0 mmol/L or more [35]. The generation of ketosis is closely related to the negative energy balance and abnormal fat mobilization in the body, which leads to enhanced ketogenesis. Ketogenesis plays an important role in the metabolism of cows, as the liver is able to convert fat into energy for use by the body under certain conditions when the body of cows is short of energy. However, when the fat metabolism of the body is excessive, the liver will produce large amounts of ketone bodies, and since the main components of ketone bodies are acidic substances, the large accumulation of ketone bodies often leads to an imbalance in the acid-base balance of the animal, causing acidosis, which may eventually lead to the occurrence of ketosis [36]. This experiment induced subacute ruminal acidosis by a high concentrate, which may lead to secondary ketosis due to the reduced dry matter intake, reduced number of substrates for microbial digestion, and altered ruminal microflora, resulting in the reduced digestive efficiency of the organism and ultimately exacerbating the negative energy balance in the body [37]. In addition, high concentrate-induced SARA significantly increased the plasma concentrations of NEFA and BHBA in cows, which may be due to the fact that cows under a high concentrate induction are in a negative energy balance and their body fat is mobilized as NEFA, thus increasing the risk of ketosis [38]. High concentrate-induced SARA can significantly reduce the plasma GLU levels in cows, and when the rumen is at low pH and high starch conditions, *Streptococcus bovis* ferments glucose to lactic acid, which further lowers the rumen pH and exacerbates the inflammatory response in the organism. During SARA, the plasma content of UREA in cows decreased significantly, probably due to the fact that high-concentration induction can cause local inflammatory reactions in the liver, thus reducing the utilization of nitrogen by cows.

However, compared with a HG diet, the supplementation of VE could alleviate the reduction in the plasma UREA induced by high-concentrate SARA, which may be due to the protective effect of VE on the liver, improving the utilization rate of ammonia in the body, thus promoting the detoxification function of the liver. In addition, VE has a certain regulation effect on the plasma GLU content, but the effect is not significant. VE can increase the molar ratio of propionic acid in the rumen, which is the main raw material of gluconeogenesis, leading to an increase in the blood glucose content.

Blood immunity and antioxidant indexes can reflect the health status of the body. It showed that when cows were in SARA, the levels of LPS and HIS in the blood increased [39]. It reported that the increased levels of LPS and HIS in blood could increase the levels of pro-inflammatory factors such as IL-1β, IL-6, and TNF-α in plasma. In this experiment, SARA induced by high concentration can significantly increase the contents of cytokines and inflammatory immune proteins in plasma, and significantly reduce the plasma antioxidant capacity [40]. This may be due to the fact that the reduction in the rumen pH caused by high concentrations alters the rumen fermentation pattern and oxidative stress status of the cow, thus causing changes in immunity and the metabolism [4].

However, the supplementation of VE was able to alleviate the rise in the plasma LPS, HIS, and pro-inflammatory factors caused by high concentrate-induced SARA; this might be due to VE having an antioxidant effect, protecting the rumen bacteria and epithelial cell membranes from oxygen free radical attacks, guaranteeing the rumen epithelial cell membrane integrity, reducing the migration of rumen LPS and HIS, thus alleviating the inflammatory response of the organism. In addition, VE also improves the plasma antioxidant capacity, probably due to its ability to release hydrogen atoms on hydroxyl groups, which rapidly trap oxygen radicals and minimize the production of secondary radicals, thus reducing oxidative stress in the organism.

### 4.4. Blood Metabolites

The purine metabolism in the blood plays an important role in the anti-inflammatory process. In this experiment, compared with the HG diet, the expression levels of guanine, hypoxanthine, guanine nucleoside, and inosine were significantly up-regulated in the HGE diet, and 3’-AMP also showed a trend of up-regulation. The purine metabolic pathway Hypoxanthine Phosphoribosyl Transferase (HPRT) and Adenine Phosphoribosyltran-sferase (APRT) help reclaim hypoxanthine, inosine, and adenine and use them as substrates to synthesize 3’-AMP. 3’-AMP is an important regulatory factor, which can regulate the metabolism and inflammatory response [41]. This further suggests that the regulation of VE can promote the purine metabolism in the blood of the body, thus enhancing the anti-inflammatory effect of the body. The expression of L-cysteine in the blood of SARA cows was significantly up-regulated by a high-grain diet. Cysteine is a precursor of glutathione and acetyl-CoA. As blood cysteine levels rise, so do the glutathione and acetyl-CoA levels. Acetyl- CoA participates in the tricarboxylic acid cycle, which also explains the increased glucose metabolism in the body during a high-concentrate induction. Glutathione can regulate the oxidative stress and lipid peroxidation of the animal body and has a strong antioxidant effect. This indicates that the body can enhance the antioxidant effect through the cysteine metabolism pathway. However, the expression of L-cysteine in the blood of cows was significantly down-regulated by the supplementation of VE, which may be because VE, as an antioxidant, can inhibit the expression of other antioxidants in the body. In addition, the co-metabolites of the CON-HG and HG-HGE were significantly enriched in the ABC transporter pathway. The ABC transporter is widely distributed in animals, and its main role is to transport sugars, amino acids, phospholipids, and peptides on the plasma membrane of bacteria or cells. Therefore, the ABC transporter affects the carbohydrate intake of animals, which may be the reason why the dry matter intake of cows decreased after SARA and increased after the regulation of VE.

### 4.5. Rumen Bacterial Populations

To further explore the different rumen fermentation mechanisms, we performed 16S rRNA gene sequencing to reveal the disparity of microbial communities in the rumen between the HG and HGE diets. In this experiment, the Shannon index in the HG diet was significantly lower than that in the CON diet, which indicated that SARA induced by a high concentration could reduce the diversity of the rumen microflora of cows. The decrease in the rumen microbial diversity in SARA cows was mainly due to the death and disintegration of Gram-negative bacteria caused by a low rumen pH value. Shannon and Simpson in the HGE diet were significantly lower than those in the CON and HG diets, suggesting that the regulation of VE at 12,000 IU/head per day could reduce the bacterial diversity in the rumen, which might be caused by the bacteriostasis effect of VE [42].

At the phylum level, in this study, SARA induced by a high concentration can cause a significant decrease in the relative abundance of *Firmicutes* in cows, which is inconsistent with the result of studies [43]. This might be related to design differences, such as the concentrate to forage ratio or the adaptability of individual cows to a high-grain diet. However, the relative abundance of *Proteobacteria* in the HGE diet was significantly higher than that in the other two periods, which might be due to *Proteobacteria* being related to the antioxidant activity of VE. An increased relative abundance of *Proteobacteria* can enhance the antioxidant and anti-inflammatory capacity and promote intestinal health [44]. In addition, the relative abundance of *Spirochaetota* in the HGE diet was significantly lower than that in the HG diet, suggesting that the supplementation of high-dose VE could change the relative abundance of *Spirochaetota* and reduce the breakdown of cellulose by rumen microorganisms, thus alleviating SARA.

At the genus level, the relative abundance of *Succinivibrionaceae_UCG-001*, *Prevotella*, and *Pseudoscardovia* in the rumen in the HG and HGE diets was significantly higher than that in the CON diet. *Succinivibrionaceae_UCG-001* and *Prevotella* were mainly involved in starch degradation, which also explained the large increase in the volatile fatty acid content in the rumen induced by a high concentration. However, the relative abundance of *Rikenellaceae_RC9_gut_group* and *Ruminococcus* in the ruminate of cows were significantly downregulated during SARA induced by a high concentration, which was similar to previous research results [45]. All these indicated that the relative abundance of starch decomposition bacteria and cellulose decomposition bacteria increased and decreased in the rumen flora induced by a high concentration. A high concentrate induction also reduced the relative abundance of *Christensenellaceae_R-7_group* in the rumen. *Christensenellaceae_R-7_group* can participate in biohydrogenation, and its relative abundance is positively correlated with the rumen pH value, while it is negatively correlated with the acetic acid content [46]. This might explain the decrease in the rumen pH and increase in the acetic acid content induced by a high concentration. In addition, the regulation of VE will significantly reduce the relative abundance of *Treponema*, *Fibrobacter*, and *Olsenella* and increase the relative abundance of *Succiniclasticum*. These results suggest that VE can reduce the production of TVFA in the rumen by regulating the relative abundance of *Treponema* and *Succiniclasticum*, thus regulating the response degree of SARA.

### 4.6. Correlations between Blood Metabolome and Rumen Microbiome

*Pseudoscardovia* is an important component of Gram-positive bacteria in the rumen and plays an important role in glycolysis [47]. *Olsenella* is a Gram-positive bacterium with a strong ability to utilize carbohydrates to generate lactic acid and is a member of a broad range of lactic acid bacteria [48]. The final products of *Pseudoscardovia* and *Olsenella* were organic acids, so their relative abundance was positively correlated with the concentration of organic acids, such as 3-methyladipic acid. *Ruminococcus* is related to rumen fiber degradation. In the previous study, it was found that the high-concentrate feeding mode inhibited the activity of *Ruminococcus*. DL-o-Tyrosine and Phenylalanine are important metabolites of the phenylalanine metabolism, and phenylalanine can also participate in the tricarboxylic acid cycle through the phenylalanine metabolism and can eventually produce glucose. In this experiment, the concentrations of DL-o-Tyrosine and phenylalanine were positively correlated with the relative abundance of *Pseudoscardovia* and *Olsenella* and negatively correlated with *Ruminococcus*. The carbohydrate intake of cows in the high-concentrate feeding mode increased, the relative abundance of *Ruminococcus* decreased significantly, and the contents of DL-o-Tyrosine and Phenylalanine were significantly increased by starch-degrading bacteria such as *Prevotella* and *Succinivibrionaceae_UCG-001*. At the same time, the decrease in the rumen pH can lead to a large amount of apoptosis of Gram-negative bacteria in the rumen, and the relative abundance of Gram-positive bacteria is significantly increased, so the relative abundance of *Olsenella* and *Pseudoscardovia* is significantly increased.

In this experiment, it was found that *Succiniclasticum* had a significant positive correlation with arachidonic acid and D-Ribulose 5-phosphate by supplementing VE in high-concentrate diets. *Succiniclasticum* can ferment succinic acid and play an important role in the acid hydrolysis and acid utilization of rumen starch. It found that *Succiniclasticum* was related to the feeding conversion efficiency and energy metabolism, and the higher nutrient absorption and conversion efficiency in the rumen, the higher its relative expression abundance [49]. Arachidonic acid can be synthesized de novo from acetyl CoA via fatty acids and then produced by a series of enzymes. Arachidonic acid is involved in the pathway of unsaturated fatty acid synthesis in the body, and the significant increase in its content indicates that the lipid metabolism in the body is intensified. Pyruvate is the substrate for acetyl CoA, the beginning of fat synthesis. Pyruvate is a key intermediate product in the glucose metabolism. It can realize the mutual conversion of sugars, fats, and amino acids through acetyl CoA and tricarboxylic acid cycles [50]. D-Ribulose 5-phosphate is an intermediate of the pentose phosphate pathway. The pentose phosphate pathway allows for glucose to be dehydrogenated and decarboxylated directly without the need for glycolysis and the tricarboxylic acid cycle. Compared with the HG diet, the relative abundance of *Succiniclasticum* in the HGE diet was significantly increased, which showed that the supplementation of VE in a high-grain diet could reduce the body’s pyruvate metabolism, enhance the pathway of lipid synthesis and pentose phosphate, reduce the production of volatile fatty acids in rumen, and alleviate the SARA response.

Although good progress was made in this study in terms of VE alleviating SARA in dairy cows, due to the limited number of cows and fistulas, this is of concern for commercial dairy producers who feed their cows VE for the alleviation of high concentrate-induced SARA in daily production. In addition, this trial resulted in the cows having high ketogenic levels due to a negative energy balance, but the cows did not exhibit the symptoms associated with ketosis. This may be related to differences in the diet composition, animal physiological stage, local climate, and different feeding management methods. However, an important contribution of this study is to provide a more comprehensive understanding of high-concentrate SARA-induced interventions. Therefore, we will follow up with a large sample trial to further investigate and analyze the effect of VE on SARA.

## 5. Conclusions

In summary, SARA induced by high-concentrate diets can reduce the DMI, rumen pH, and blood antioxidant capacity of cows, disrupt the normal blood metabolism and rumen fermentation environment, and interfere with metabolic pathways such as aromatic compound degradation and ABC transporter protein synthesis, thereby reducing the performance of cows. A VE supplementation in high concentrate diets can improve the lactation performance and rumen fermentation pattern, promote the purine metabolism, amino acid metabolism, and ABC transporter protein synthesis in cows. VE can also reduce the pyruvate metabolism, enhance lipid synthesis and the pentose phosphate pathways, and reduce the production of volatile fatty acids in the rumen, thus helping to alleviate SARA cows under a high concentration induction. Considering that the cows in this trial were established at high ketone levels, we can assume that vitamin E can alleviate SARA cows at high ketone levels. Therefore, the results of this study also clarified the role of VE in the regulation of the rumen metabolism and provided a theoretical reference for the alleviation of SARA.

## Figures and Tables

**Table 1:** Composition and nutrient levels of the experimental diets (dry matter basis).

Items	Dietary Treatment ^1^		
	CON	HG	HGE
Ingredient composition, %			
Whole plant corn silage	24.32	16.78	16.78
Alfalfa hay	13.75	9.69	9.69
Oats hay	11.45	8.32	8.32
Ground corn	17	17.12	17.12
Ground wheat	0	15.19	15.19
Ground barley	3	3.03	3.03
Soybean meal	13.41	13.5	13.5
Whole cotton seed	5.23	5.27	5.27
Wet brewer’s grains	4.93	4.14	4.14
Cane molasses	2.65	2.67	2.67
Rumen bypass fat	0.39	0.4	0.4
Premix ^2^	0.84	0.84	0.84
Calcium hydrogen phosphate	0.36	0.36	0.36
Limestone	1.12	1.13	1.13
Sodium bicarbonate	0.81	0.82	0.82
KCl	0.07	0.07	0.07
MgO	0.31	0.31	0.31
NaCl	0.36	0.36	0.36
Nutrient levels ^3^			
NEL, MJ/kg	6.67	6.97	6.97
CP	16.62	16.63	16.63
NDF	35.81	29.22	29.22
ADF	22.6	17.41	17.41
EE	4.43	4.25	4.25
Starch	21.97	31.28	31.28
Ca	0.89	0.82	0.82
P	0.39	0.41	0.41

^1^ CON = control diet, HG = high-grain diet, HGE = high vitamin E diet. ^2^ Mineral vitamin premix, each Kg premix (DM base) contains: vitamin A, 1,380,000 IU/kg; vitamin D, 386,400 IU/kg; vitamin E, 9940 IU/kg; Cu, 2400 mg/kg; Zn, 20,000 mg/kg; Mn, 15,600 mg/kg; Se, 100 mg/kg; I, 300 mg/kg; Co, 240 mg/kg. ^3^ Net energy of lactation (NEL) was a calculated value, and the others were measured values.

**Table 2:** Effects of vitamin E on the performance of subacute ruminal acidosis cows.

Item ^1^	Dietary Treatments ^2^			SEM ^3^	*p*-Value	
	CON	HG	HGE		CON vs. HG	HG vs. HGE
DMI, kg/d	21.4	19.4	22.2	0.28	<0.01	<0.01
Milk yield, kg/d	32.9	29.4	32.9	1.16	0.23	0.24
3.5%FCM, kg/d	36.6	26.9	32.9	1.20	<0.01	<0.01
ECM, kg/d	36.0	28.0	33.3	1.09	<0.01	0.02
Feed efficiency	1.7	1.4	1.5	0.05	<0.01	0.32
Milk fat, %	4.2	3.2	3.6	0.2	0.05	0.33
Milk protein, %	3.1	3.3	3.2	0.28	<0.01	0.12
Lactose, %	4.5	4.8	4.6	0.41	0.01	0.13
Total milk solid, %	12.9	12.4	12.5	0.24	0.44	0.87
Milk fat yield, kg/d	1.4	0.9	1.2	0.06	<0.01	0.02
Milk protein yield, kg/d	1.0	1.0	1.1	0.04	0.60	0.43
Lactose yield, kg/d	1.5	1.4	1.5	0.05	0.60	0.42
Total solids production, kg/d	4.2	3.6	4.1	0.13	0.03	0.11
MUN, mg/dL	13.7	11.3	11.7	0.43	0.02	0.68
NUE, g/g	0.3	0.3	0.3	0.01	0.59	0.55

^1^ MUN = milk urea nitrogen; NUE = nitrogen use efficiency. ^2^ CON = control diet, HG = high-grain diet, HGE = high vitamin E diet. ^3^ SEM standard error of the mean.

**Table 3:** Effects of vitamin E on milk’s antioxidant capacity induced by high concentration in subacute ruminal acidosis cows.

Item	Dietary Treatments ^1^			SEM ^2^	*p*-Value	
	CON	HG	HGE		CON vs. HG	HG vs. HGE
T-AOC, mM	0.8	0.7	0.8	0.01	<0.01	0.03
MDA, nmol/mL	5.0	8.0	6.1	0.19	<0.01	<0.01
LPS, U/L	398.7	397.2	444.7	10.46	0.95	0.06
SOD, U/mL	148.7	98.5	128.1	3.30	<0.01	<0.01
CAT, U/mL	100.9	75.9	90.0	2.12	<0.01	<0.01
GSH-Px, U/mL	845.8	657	740.7	15.86	0.05	0.01

^1^ CON = control diet, HG = high-grain diet, HGE = high vitamin E diet. ^2^ SEM standard error of the mean.

**Table 4:** Effects of vitamin E on rumen pH and abnormal metabolites induced by high concentration in subacute ruminal acidosis cows.

Item ^1^	Dietary Treatments ^2^			SEM ^3^	*p*-Value	
	CON	HG	HGE		CON vs. HG	HG vs. HGE
Maximum ruminal pH	6.9	6.5	6.7	0.07	0.01	0.26
Minimum ruminal pH	6.0	5.5	5.6	0.08	0.01	0.39
Mean ruminal pH	6.4	6.1	6.2	0.10	0.23	0.72
0 h	6.9	6.5	6.7	0.07	0.01	0.26
3 h	6.4	5.7	5.8	0.09	<0.01	0.27
6 h	6.0	5.5	5.6	0.08	0.01	0.39
9 h	6.4	6.1	6.0	0.09	0.26	0.70
12 h	6.6	6.5	6.6	0.08	0.47	0.46
NH_3_-N, mg/100 mL	11.1	8.4	11.7	0.58	0.03	0.01
LPS, EU/mL	19.7	26.8	20.0	0.56	<0.01	<0.01
HIS, ng/mL	20.6	25.1	22.1	0.39	<0.01	<0.01
LA, mmol/L	0.1	0.1	0.1	0.01	0.29	0.47

^1^ LPS = lipopolysaccharide; HIS = histamine; LA = lactic acid. ^2^ CON = control diet, HG = high-grain diet, HGE = high vitamin E diet. ^3^ SEM standard error of the mean.

**Table 5:** Effects of vitamin E on volatile fatty acids in rumen induced by high concentration in subacute ruminal acidosis cows.

Item ^1^	Dietary Treatments ^2^			SEM ^3^	*p*-Value	
	CON	HG	HGE		CON vs. HG	HG vs. HGE
Acetate, ng/uL	54.9	66.6	60.5	1.48	<0.01	<0.01
Propionate, ng/uL	17.0	28.8	26.4	2.41	0.04	0.63
Butyrate, ng/uL	12.6	19.9	14.4	1.26	0.01	0.04
Isobutyrate, ng/uL	0.8	0.9	1.0	0.04	0.54	0.10
Valerate, ng/uL	1.4	2.4	2.1	0.17	0.01	0.49
Isovalerate, ng/uL	1.2	1.5	1.9	0.15	0.48	0.24
TVFA, ng/uL	87.9	120.0	106.4	4.21	<0.01	0.01
A/P	3.2	2.5	2.4	0.19	0.13	0.88
Molar ratio, %						
Acetate	62.5	55.6	57.1	1.19	0.01	0.52
Propionate	19.3	23.8	24.5	1.54	0.26	0.85
Butyrate	14.4	16.7	13.7	0.86	0.30	0.18

^1^ TVFA = total volatile fatty acids; A/P = acetate/propionate. ^2^ CON = control diet, HG = high-grain diet, HGE = high vitamin E diet. ^3^ SEM standard error of the mean.

**Table 6:** Effects of vitamin E on plasma biochemical indices induced by high concentration in subacute ruminal acidosis cows.

Item ^1^	Dietary Treatments ^2^			SEM ^3^	*p*-Value	
	CON	HG	HGE		CON vs. HG	HG vs. HGE
ALB, g/L	48.5	48.5	48.5	0.33	0.96	0.99
TP, g/L	78.1	77.6	78.2	0.58	0.72	0.65
ALB/GLB	1.7	1.7	1.6	0.01	0.40	0.28
GLB, g/L	29.6	29.1	29.7	0.31	0.47	0.40
GLU, mmol/L	4.3	3.9	4.0	0.08	0.02	0.67
LDH, U/L	775.2	789.7	820.2	16.26	0.72	0.45
TC, mmol/L	3.9	3.4	3.5	0.09	0.03	0.71
TG, mmol/L	0.1	0.1	0.1	0.002	0.85	0.09
UREA, mmol/L	4.3	3.3	4.5	0.11	<0.01	<0.01
NEFA, mmol/L	0.3	0.4	0.4	0.005	0.01	0.83
BHBA, mmol/L	2.3	2.6	2.6	0.06	0.01	0.82
INS, μIU/mL	36.8	30.7	39.8	2.71	0.36	0.18
GC, pg/mL	194.6	205.8	185.2	3.67	0.21	0.02

^1^ INS = insulin concentrations; GC = glucagon; LDH = lactate dehydrogenase. ^2^ CON = control diet, HG = high-grain diet, HGE = high vitamin E diet. ^3^ SEM standard error of the mean.

**Table 7:** Effects of vitamin E on plasma immune indices induced by high concentration in subacute ruminal acidosis cows.

Item ^1^	Dietary Treatments ^2^			SEM ^3^	*p*-Value	
	CON	HG	HGE		CON vs. HG	HG vs. HGE
IL-1β, pg/mL	185.9	319.6	267.8	10.11	<0.01	0.01
IL-6, pg/mL	134.8	171.8	141.9	3.36	<0.01	<0.01
IL-10, pg/mL	46.2	49.5	46.6	1.31	0.33	0.39
TLR4, ng/mL	10.6	10.6	10.0	0.39	0.97	0.57
TNF-α, pg/mL	130.8	178.6	157.7	3.67	<0.01	<0.01
Hp, ng/mL	260.6	335.5	313.8	6.21	<0.01	0.07
SAA, ug/mL	8.8	10.3	9.2	0.19	<0.01	0.01
CRP, mg/L	7.2	9.1	8.4	0.18	<0.01	0.06
LBP, ng/mL	578.4	582.2	628.0	14.84	0.92	0.21
Endotoxin, EU/mL	12.0	17.0	13.6	0.32	<0.01	<0.01
HIS, ng/mL	11.6	15.7	13.5	0.25	<0.01	<0.01
LA, mmol/L	0.2	0.2	0.2	0.01	0.13	0.69

^1^ TLR4 = toll-like receptor 4; Hp = haptoglobin; SAA = serum amyloid; LBP = LPS-binding protein; HIS = histamine; LA = lactic Acid. ^2^ CON = control diet, HG = high-grain diet, HGE = high vitamin E diet. ^3^ SEM standard error of the mean.

**Table 8:** Effects of vitamin E on plasma antioxidant capacity induced by high concentration in subacute ruminal acidosis cows.

Item	Dietary Treatments ^1^			SEM ^2^	*p*-Value	
	CON	HG	HGE		CON vs. HG	HG vs. HGE
T-AOC, mM	0.7	0.6	0.6	0.01	<0.01	0.98
MDA, nmol/mL	10.7	14.1	12.3	0.31	<0.01	0.01
LPS, U/L	905.0	949.4	889.5	19.69	0.36	0.22
SOD, U/mL	330.1	269.0	304.6	5.02	<0.01	<0.01
CAT, U/mL	243.0	207.3	220.0	3.39	<0.01	0.08
GSH-Px, U/mL	1153.3	868.9	1037.7	22.87	<0.01	<0.01

^1^ CON = control diet, HG = high-grain diet, HGE = high vitamin E diet. ^2^ SEM standard error of the mean.

**Table 9:** Effects of vitamin E on rumen OTUS diversity induced by high concentration in subacute ruminal acidosis cows.

Item ^1^	Dietary Treatments ^2^			SEM ^3^	*p*-Value	
	CON	HG	HGE		CON vs. HG	HG vs. HGE
Good’s_coverage/%	99.6	99.6	99.6	<0.01	1	0.55
OTU	1471.4	1385.0	1311.9	21.43	0.08	0.14
ACE	1573.4	1489.3	1422.5	24.41	0.10	0.19
Chao1	1568.8	1480.8	1412.0	24.52	0.08	0.16
Shannon	8.6	8.1	7.4	0.14	<0.01	<0.01
Simpson	1.0	1.0	1.0	<0.01	0.08	<0.01

^1^ OTU = operational taxonomic unit. ^2^ CON = control diet, HG = high-grain diet, HGE = high vitamin E diet. ^3^ SEM standard error of the mean.

**Table 10:** Analysis of differences during MRPP.

Treatments	A ^1^	Observed-Delta ^2^	Expected-Delta ^3^	Significance ^4^
CON-HG	0.1	0.5	0.5	<0.01
HG-HGE	0.1	0.4	0.4	<0.01

^1^ A represents the comparison of the differences between and within groups. ^2^ Observed-delta represents the difference within the group, the smaller the value, the smaller the difference within the group. ^3^ Expected-delta indicates the difference between groups. A larger value indicates a larger difference between groups. ^4^ Significance means the significance of the difference.

## Data Availability

The data presented in this study are available on request from the corresponding author.

## Supplementary Materials

The following supporting information can be downloaded at: https://www.mdpi.com/article/10.3390/ani13030486/s1. Table S1: Major differential metabolites in serum; Table S2: Common differential metabolites of CON-HG and HG-HGE; Table S3: Effects of vitamin E on rumen microflora phylum levels induced by high concentrate in SARA cows; Table S4: Effects of vitamin E on genus level of rumen microflora induced by high concentrate in SARA cows.
