# Supplementary material for: High-Dose Vitamin E Supplementation Can Alleviate the Negative Effect of Subacute Ruminal Acidosis in Dairy Cows
Source: Animals (Basel). 2023 Jan 31;13(3):486. doi: 10.3390/ani13030486 (PMC9913405; doi:10.3390/ani13030486)
Supplement: Supplementary file 1 [file animals-13-00486-s001.zip › animals-2069451-supplementary.pdf]

**Table S1.** Major differential metabolites in serum

| Metabolite | FC[1] | *p*[2] | VIP[3] | Up/Down |
|---|---|---|---|---|
| CON-HG | | | | |
| Salicylic acid | 2.44 | <0.01 | 1.61 | up |
| Catechol | 1.79 | 0.01 | 1.30 | up |
| 4-Methylcatechol | 2.06 | 0.03 | 1.49 | up |
| 4-Methylphenol | 1.82 | <0.01 | 1.63 | up |
| o-Cresol | 1.55 | 0.01 | 1.60 | up |
| HG.vs.HGE | | | | |
| Guanine | 0.40 | 0.03 | 1.78 | down |
| Hypoxanthine | 0.42 | 0.04 | 1.71 | down |
| Guanosine | 0.04 | <0.01 | 2.78 | down |
| Inosine | 0.53 | 0.04 | 1.66 | down |
| 3'-AMP | 0.64 | 0.06 | 1.15 | down |

[1] FC = fold change is the ratio of the mean values of all biological repeated quantitative values of each metabolite in the test period.

[2] *p*-value represents the significance level of difference between the two groups

[3] VIP refers to the variable projection importance of the first principal component of the PLS-DA model

**Table S2.** Common differential metabolites of CON-HG and HG-HGE

| Metabolite | CON-HG | | | HG-HGE | | |
|---|---|---|---|---|---|---|
| | FC[1] | *p*[2] | VIP[3] | FC | *p* | VIP |
| N-Phenylacetylglutamine | 2.92 | <0.01 | 1.82 | 0.66 | 0.03 | 1.08 |
| 1-(4-benzylpiperazino)-2-(pyridin-2-ylamino)propan-1-one | 0.61 | <0.01 | 1.30 | 0.62 | <0.01 | 1.45 |
| 4-Methylcatechol | 2.06 | 0.03 | 1.49 | 0.46 | 0.02 | 2.06 |
| Choline | 1.79 | 0.03 | 1.49 | 0.58 | 0.02 | 1.89 |
| 3-Methyladipic acid | 0.37 | <0.01 | 1.87 | 2.05 | 0.01 | 2.02 |
| 4-Methylphenol | 1.82 | <0.01 | 1.63 | 0.50 | <0.01 | 2.49 |
| 2-Mercaptobenzothiazole | 0.04 | <0.01 | 2.28 | 2.46 | 0.04 | 1.03 |
| P-Coumaroyl Agmatine | 2.17 | 0.01 | 1.26 | 1.51 | 0.02 | 1.12 |
| N-{4-[(2R,3R)-3-(Hydroxymethyl)-5-oxo-2-morpholinyl]phenyl}acetamide | 2.88 | 0.02 | 1.60 | 0.51 | <0.01 | 1.63 |
| DL-o-Tyrosine | 0.20 | <0.01 | 1.91 | 3.18 | 0.02 | 1.75 |
| 2-[(3-methylbenzo[b]thiophen-2-yl)carbonyl]benzoic acid | 2.76 | 0.02 | 1.47 | 0.33 | 0.04 | 1.77 |
| Oxoadipic Acid | 0.30 | <0.01 | 1.82 | 2.83 | 0.01 | 2.13 |
| Ureidoisobutyric Acid | 0.15 | <0.01 | 2.08 | 2.95 | 0.01 | 1.53 |
| Stearic acid | 2.03 | <0.01 | 1.56 | 0.51 | 0.05 | 1.64 |
| D-Phenylalanine | 0.16 | <0.01 | 2.08 | 3.02 | 0.02 | 1.49 |
| Cystine | 0.41 | <0.01 | 2.03 | 2.17 | <0.01 | 2.18 |
| 11-Epiprostaglandin E1 | 1.60 | 0.04 | 1.25 | 0.57 | 0.04 | 1.87 |
| trans-3-Hydroxycotinine | 0.27 | <0.01 | 2.38 | 1.50 | 0.01 | 1.05 |
| L-Tryptophan | 0.23 | 0.01 | 1.70 | 3.42 | 0.02 | 1.92 |
| 11(Z),14(Z),17(Z)-Eicosatrienoic acid | 0.50 | <0.01 | 1.71 | 2.11 | <0.01 | 2.45 |
| LPC 10:0 | 0.52 | 0.02 | 1.03 | 3.10 | <0.01 | 2.51 |
| octadec-9-ynoic acid | 0.64 | 0.01 | 1.12 | 2.36 | <0.01 | 2.95 |
| (+)-alpha-Lipoic acid | 0.45 | 0.01 | 1.57 | 1.89 | 0.03 | 1.74 |
| Lysopa 18:0 | 1.81 | <0.01 | 1.22 | 0.35 | <0.01 | 2.56 |
| LPC 13:0 | 0.50 | 0.03 | 1.23 | 1.93 | 0.03 | 1.66 |
| Ethyl 3-methyl-5-[(phenoxycarbonyl)amino]isothiazole-4-carboxylate | 1.53 | 0.03 | 1.43 | 0.56 | 0.01 | 2.33 |
| 3,8,9-trihydroxy-10-propyl-3,4,5,8,9,10-hexahydro-2H-oxecin-2-one | 0.44 | 0.02 | 1.38 | 2.26 | 0.02 | 2.00 |
| NNK | 2.21 | <0.01 | 1.11 | 1.98 | <0.01 | 1.54 |
| L-Glutathione (reduced) | 1.74 | <0.01 | 1.77 | 0.63 | <0.01 | 2.12 |

[1] FC is the ratio of the mean values of all biological repeated quantitative values of each metabolite in the test period.

[2] *p*-value represents the significance level of difference between the two groups

[3] VIP refers to the variable projection importance of the first principal component of the PLS-DA model

**Table S3.** Effects of vitamin E on rumen microflora phylum levels induced by high concentration in subacute ruminal acidosis cows

| Phylum | Dietary treatments[1] | | | SEM[2] | *p*-Value | |
|---|---|---|---|---|---|---|
| | CON | HG | HGE | | CON vs. HG | HG vs. HGE |
| *Bacteroidota* | 48.18 | 48.13 | 45.68 | 0.75 | 0.98 | 0.20 |
| *Firmicutes* | 43.21 | 35.98 | 32.70 | 1.64 | 0.04 | 0.30 |
| *Proteobacteria* | 1.12 | 5.10 | 13.44 | 1.56 | 0.06 | <0.01 |
| *Spirochaetota* | 1.66 | 2.14 | 1.02 | 0.22 | 0.34 | 0.04 |
| *Actinobacteriota* | 1.03 | 2.72 | 1.83 | 0.29 | 0.01 | 0.15 |
| *Fibrobacterota* | 0.88 | 1.29 | 0.46 | 0.17 | 0.29 | 0.04 |
| *Cyanobacteria* | 0.20 | 0.61 | 0.46 | 0.12 | 0.18 | 0.62 |
| *Euryarchaeota* | 1.04 | 0.77 | 0.59 | 0.09 | 0.18 | 0.37 |
| *unidentified_Bacteria* | 0.91 | 0.91 | 0.92 | 0.02 | 0.96 | 0.96 |
| *Desulfobacterota* | 0.11 | 0.09 | 0.11 | 0.01 | 0.44 | 0.58 |

[1] CON = Control stage, HG = High-grain diet, HGE = High vitamin E diet.

[2] SEM standard error of the mean.

**Table S4.** Effects of vitamin E on genus level of rumen microflora induced by high concentration in subacute ruminal acidosis cows

| Genus | Dietary treatments[1] | | | SEM[2] | *p*-Value | |
|---|---|---|---|---|---|---|
| | CON | HG | HGE | | CON vs. HG | HG vs. HGE |
| *Prevotella* | 19.45 | 25.12 | 24.55 | 1.01 | 0.01 | 0.78 |
| *Succinivibrionaceae_UCG-001* | 0.33 | 3.94 | 10.55 | 1.23 | 0.01 | <0.01 |
| *Rikenellaceae_RC9_gut_group* | 7.69 | 4.20 | 3.63 | 0.54 | <0.01 | 0.43 |
| *Treponema* | 1.61 | 2.11 | 0.98 | 0.22 | 0.32 | 0.04 |
| *Succiniclasticum* | 3.62 | 2.50 | 5.30 | 0.49 | 0.29 | 0.02 |
| *Ruminococcus* | 7.39 | 1.29 | 3.88 | 0.47 | <0.01 | 0.38 |
| *Fibrobacter* | 0.88 | 0.61 | 0.46 | 0.17 | 0.30 | 0.04 |
| *Olsenella* | 0.41 | 1.28 | 0.35 | 0.16 | 0.01 | 0.01 |
| *Pseudoscardovia* | 0.08 | 0.92 | 0.86 | 0.16 | 0.03 | 0.87 |
| *unidentified_Chloroplast* | 0.12 | 0.57 | 0.36 | 0.12 | 0.15 | 0.48 |
| *Succinivibrionaceae_UCG-002* | 0.41 | 0.51 | 1.08 | 0.12 | 0.71 | 0.06 |
| *Christensenellaceae_R-7_group* | 3.92 | 1.93 | 1.29 | 0.32 | <0.01 | 0.06 |
| *Acetitomaculum* | 3.46 | 2.61 | 1.93 | 0.20 | 0.02 | 0.04 |
| *Prevotellaceae_UCG-001* | 2.10 | 1.88 | 1.93 | 0.11 | 0.44 | 0.88 |
| *unidentified_Mitochondria* | 0.00 | 0.02 | 0.42 | 0.09 | 0.93 | 0.07 |
| *NK4A214_group* | 2.79 | 1.51 | 1.09 | 0.21 | <0.01 | 0.07 |
| *Selenomonas* | 0.57 | 1.34 | 0.73 | 0.10 | <0.01 | <0.01 |
| *Lachnospiraceae_NK3A20_group* | 1.92 | 1.88 | 1.09 | 0.12 | 0.82 | <0.01 |
| *Saccharofermentans* | 2.17 | 1.47 | 1.18 | 0.14 | 0.01 | 0.18 |
| *Prevotellaceae_NK3B31_group* | 1.26 | 1.31 | 0.87 | 0.06 | 0.52 | <0.01 |
| *CAG-352* | 0.41 | 0.19 | 0.39 | 0.04 | 0.02 | 0.03 |
| *Methanobrevibacter* | 0.90 | 0.70 | 0.55 | 0.07 | 0.25 | 0.42 |
| *Bifidobacterium* | 0.28 | 0.35 | 0.37 | 0.03 | 0.47 | 0.82 |
| *Dialister* | 0.10 | 0.56 | 0.21 | 0.06 | <0.01 | <0.01 |
| *Shuttleworthia* | 0.37 | 0.73 | 0.79 | 0.07 | 0.01 | 0.63 |
| *Ruminobacter* | 0.21 | 0.34 | 0.20 | 0.03 | 0.09 | 0.06 |
| *Enterobacter* | 0.00 | 0.00 | 0.08 | 0.03 | 0.98 | 0.25 |
| *Erysipelotrichaceae_UCG-007* | 0.03 | 0.14 | 0.29 | 0.04 | 0.15 | 0.04 |
| *Butyrivibrio* | 1.10 | 0.81 | 0.49 | 0.07 | <0.01 | <0.01 |
| *UCG-005* | 1.14 | 0.59 | 0.49 | 0.09 | <0.01 | 0.33 |
| *Others* | 35.27 | 34.77 | 33.64 | 0.34 | 0.52 | 0.15 |

[1] CON = Control stage, HG = High-grain diet, HGE = High vitamin E diet.

[2] SEM standard error of the mean.
